# Supplementary material for: Binary Combinations of Essential Oils: Antibacterial Activity Against Staphylococcus aureus, and Antioxidant and Anti-Inflammatory Properties
Source: Molecules. 2025 Jan 21;30(3):438. doi: 10.3390/molecules30030438 (PMC11820965; doi:10.3390/molecules30030438)
Supplement: Supplementary file 1 [file molecules-30-00438-s001.zip › molecules-3254662-supplementary.pdf]

Supplementary Material

Binary Combinations of Essential Oils: Antibacterial Activity Against *Staphylococcus aureus*, and Antioxidant and Anti-Inflammatory Properties

Clara Naccari [1], Giovanna Ginestra [2,*], Nicola Micale [2,*], Ernesto Palma [1,3], Benedetta Galletta [2,4], Rosaria Costa [5], Rossella Vadalà [5], Antonia Nostro [2] and Mariateresa Cristani [2]

[1] Dipartimento di Scienze della Salute, Università "Magna Græcia" di Catanzaro, Viale Europa, 88100 Catanzaro, Italy; c.naccari@unicz.it (C.N.); palma@unicz.it (E.P.)
[2] Dipartimento di Scienze Chimiche, Biologiche, Farmaceutiche ed Ambientali, Università degli Studi di Messina, Viale F. Stagno D'Alcontres 31, 98166 Messina, Italy; benegalletta@yahoo.it (B.G.); anostro@unime.it (A.N.); mcristani@unime.it (M.C.)
[3] CIS—Centro Servizio Interdipartimentale—IRC-FSH "Centro di Ricerche Farmacologiche, Sicurezza degli Alimenti e Salute ad Alto Contenuto Tecnologico", Università "Magna Græcia" di Catanzaro, Viale Europa, 88100 Catanzaro, Italy
[4] Fondazione "Prof. Antonio Imbesi", Università degli Studi di Messina, Piazza Pugliatti 1, 98122 Messina, Italy
[5] Dipartimento di Scienze Biomediche, Odontoiatriche e delle Immagini Morfologiche e Funzionali, Università degli Studi di Messina, Via Consolare Valeria, 98100 Messina, Italy; costar@unime.it (R.C.); rvadala@unime.it (R.V.)
* Correspondence: gginestra@unime.it (G.G.); nmicale@unime.it (N.M.)

Figure S1. GC-MS chromatograms of (a) *Citrus aurantium* L. var. *amara* L., (b) *Cistus ladaniferus* L., (c) *Juniperus communis* L. and (d) *Origanum vulgare* L. EOs. Peak top numbers refer to Table S1.

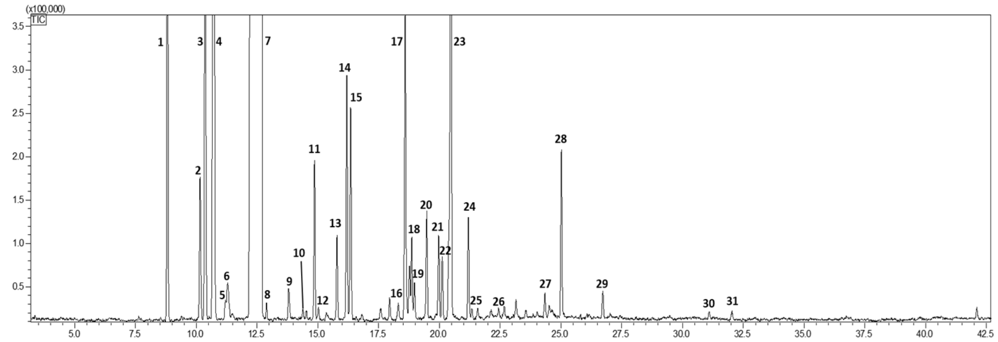

(a) *Citrus aurantium* L. var. *amara*

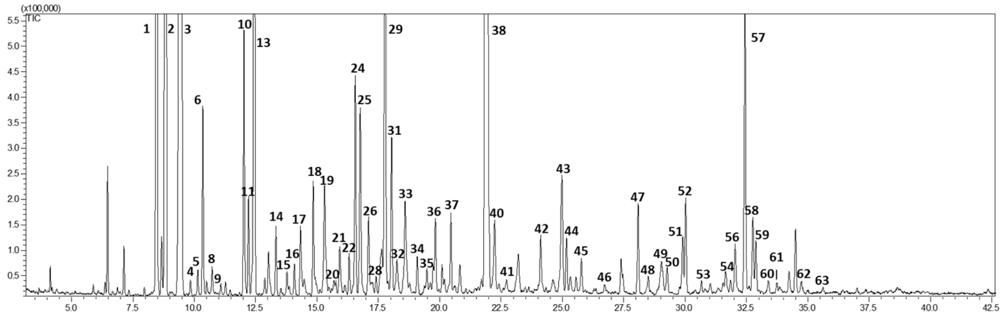

(b) *Cistus ladaniferus* L.

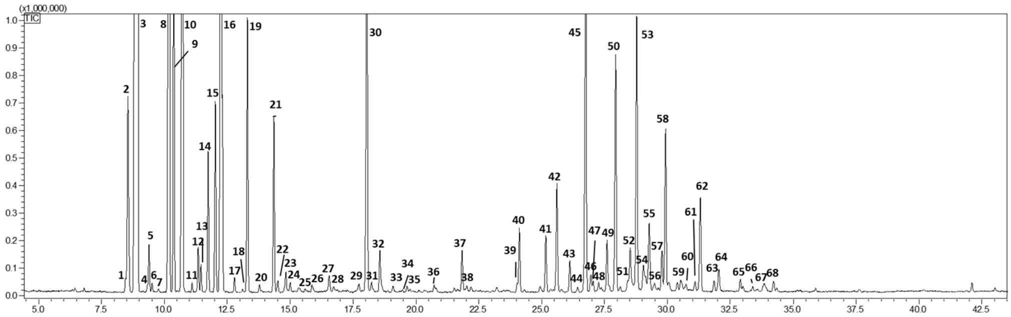

(c) *Juniperus communis* L.

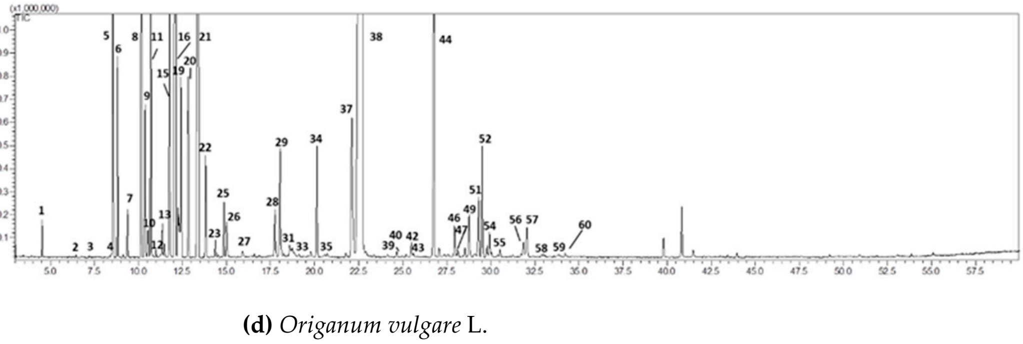

(d) *Origanum vulgare* L.

Table S1. Volatile fingerprints obtained by means of GC-MS analyses of the four EOs under investigation. (a) *Citrus aurantium* L. var. *amara,* (b) *Cistus ladaniferus* L., (c) *Juniperus communis* L. and (d) *Origanum vulgare* L. Peak top numbers refer to Table S1. RI_{exp}: experimental retention index measured on SLB-5ms column; RI_{pub}: retention index reported in mass spectral libraries.

(a) *Citrus aurantium* L. var. amara.

Peak#	Compound	RI exp	RI pub	Area (%) Mean (n = 3)	std.dev
**1**	α-pinene	930	932	0.88	0.03
**2**	sabinene	971	972	0.26	0.02
**3**	β-pinene	973	974	1.12	0.13
**4**	myrcene	990	991	3.45	0.11
**5**	octanal	995	998	0.02	0.01
**6**	p-mentha-1(7),8-diene	1001	1003	0.10	0.02
**7**	limonene	1027	1030	87.87	0.55
**8**	(E)-β-ocimene	1042	1044	0.03	0.01
**9**	octanol	1060	1063	0.06	0.01
**10**	terpinolene	1084	1086	0.03	0.01
**11**	linalool	1095	1095	0.30	0.03
**12**	nonanal	1098	1100	0.03	0.01
**13**	trans-p-2,8-menthadien-1-ol	1120	1122	0.14	0.02
**14**	cis-limonene oxide	1149	1152	0.45	0.02
**15**	trans-limonene oxide	1158	1160	0.34	0.03
**16**	trans-isocarveol	1187	1189	0.04	0.01
**17**	α-terpineol	1192	1195	0.59	0.03
**18**	decanal	1198	1201	0.12	0.02
**19**	octyl acetate	1207	1211	0.06	0.01
**20**	trans-carveol	1213	1215	0.20	0.03
**21**	cis-carveol	1224	1226	0.16	0.01
**22**	neral	1233	1235	0.11	0.01
**23**	linalyl acetate	1250	1254	1.84	0.11
**24**	geranial	1261	1264	0.18	0.02
**25**	perillaldehyde	1275	1278	0.02	0.00
**26**	perilla alcohol	1295	1299	0.03	0.00
**27**	neryl acetate	1356	1359	0.06	0.01
**28**	geranyl acetate	1378	1379	0.27	0.03
**29**	(E)-caryophyllene	1416	1417	0.06	0.01
**30**	(E)-nerolidol	1561	1561	0.02	0.00
**31**	caryophyllene oxide	1580	1582	0.02	0.01
				**98.84**	**0.58**

(b) *Cistus ladaniferus* L.

Peak#	Compound	RI exp	RI pub	Area (%) Mean (n = 3)	std.dev
**1**	tricyclene	920	921	5.08	0.13
**2**	α-pinene	932	932	13.68	0.31
**3**	camphene	947	946	37.04	0.15

**4**	2-methyl-1-hepten-6-one	947	958	0.05	0.01
**5**	sabinene	970	972	0.08	0.01
**6**	β-pinene	975	974	0.52	0.03
**7**	6-methyl-5-hepten-2-one	989	986	0.05	0.01
**8**	trans-dehydroxylinalool oxide	992	991	0.12	0.02
**9**	cis-dehydroxylinalool oxide	1008	1006	0.06	0.00
**10**	p-cymene	1023	1025	0.72	0.05
**11**	limonene	1029	1030	0.30	0.02
**12**	1,8-cineole	1031	1031	0.04	0.00
**13**	2,2,6-trimethylcyclohexanone	1036	1035	3.95	0.10
**14**	seudenone	1057	1055	0.18	0.04
**15**	cis-linalool oxide	1072	1069	0.08	0.01
**16**	2-methylcyclopentanone	1078	1075	0.12	0.01
**17**	camphenilone	1082	1078	0.29	0.03
**18**	linalool	1098	1095	0.31	0.05
**19**	3,4-dimethylcyclohexanol	1108	1105	0.51	0.03
**20**	exo-fenchol	1121	1118	0.05	0.00
**21**	α-campholenal	1129	1126	0.23	0.02
**22**	3-nonen-2-one	1142	1137	0.15	0.01
**23**	nopinone	1145	1139	0.04	0.01
**24**	trans-pinocarveol	1147	1141	0.77	0.08
**25**	camphor	1149	1141	0.73	0.03
**26**	camphene hydrate	1155	1156	0.31	0.04
**27**	trans-pinocamphone	1158	1158	0.05	0.00
**28**	isoborneol	1166	1165	0.08	0.01
**29**	borneol	1170	1173	1.65	0.16
**30**	cis-pinocamphone	1178	1176	0.04	0.01
**31**	terpinen-4-ol	1182	1180	0.60	0.08
**32**	p-cymen-8-ol	1193	1189	0.18	0.03
**33**	α-terpineol + myrtenal	1197	1195	0.58	0.09
**34**	verbenone	1213	1208	0.15	0.01
**35**	trans-carveol	1228	1223	0.09	0.01
**36**	isobornyl formate	1235	1235	0.30	0.03
**37**	linalyl acetate	1256	1254	0.25	0.03
**38**	bornyl acetate	1290	1287	21.93	0.15
**39**	isobornyl acetate	1293	1287	0.09	0.01
**40**	trans-pinocarvyl acetate	1299	1296	0.28	0.02
**41**	cis-pinocarvyl acetate	1308	1311	0.06	0.01
**42**	α-cubebene	1345	1349	0.19	0.05
**43**	cyclosativene	1365	1367	0.57	0.04
**44**	α-copaene	1374	1374	0.17	0.03
**45**	sativene	1386	1390	0.14	0.01
**46**	(E)-caryophyllene	1415	1417	0.03	0.00
**47**	alloaromadendrene	1462	1458	0.27	0.05
**48**	γ-muurolene	1477	1478	0.08	0.01
**49**	viridiflorene	1493	1496	0.22	0.01
**50**	α-muurolene	1502	1500	0.14	0.02
**51**	δ–cadinene	1516	1518	0.25	0.02
**52**	trans-calamenene	1522	1521	0.35	0.03
**53**	α-calacorene	1542	1544	0.06	0.01
**54**	palustrol	1569	1567	0.14	0.02

**55**	spathulenol	1572	1577	0.05	0.02
**56**	caryophyllene oxide	1581	1582	0.20	0.02
**57**	viridiflorol	1589	1592	1.03	0.06
**58**	ledol	1596	1602	0.26	0.03
**59**	copaborneol	1615	1613	0.27	0.04
**60**	1-epicubenol	1625	1627	0.06	0.00
**61**	α-cadinol	1638	1641	0.05	0.01
**62**	cadalene	1672	1675	0.05	0.01
**63**	10-nor-Calamenen-10-one	1699	1702	0.03	0.01
	**TOTAL**			**96.41**	**0.45**

c) *Juniperus communis* L.

**Peak#**	**Compound**	**RI $_{exp}$**	**RI $_{pub}$**	**Area (%) Mean (n = 3)**	**std.dev**
**1**	tricyclene	920	921	0.07	0.01
**2**	α-thujene	923	924	1.35	0.08
**3**	α-pinene	932	932	42.01	0.12
**4**	α-fenchene	945	948	0.02	0.00
**5**	camphene	947	946	0.26	0.03
**6**	thuja-2,4(10)-diene	955	953	0.04	0.01
**7**	verbenene	960	961	0.02	0.01
**8**	sabinene	970	972	11.66	0.12
**9**	β-pinene	973	974	2.38	0.27
**10**	myrcene	990	991	10.72	0.23
**11**	δ–2-carene	998	1000	0.05	0.01
**12**	α-phellandrene	1001	1002	0.26	0.02
**13**	δ–3-carene	1005	1008	0.17	0.02
**14**	α-terpinene	1015	1018	0.89	0.08
**15**	p-cymene	1023	1025	1.04	0.11
**16**	limonene	1029	1030	6.36	0.17
**17**	(E)-β-ocimene	1043	1044	0.10	0.02
**18**	pentyl isobutyrate	1048	1049	0.02	0.01
**19**	γ-terpinene	1054	1054	1.65	0.12
**20**	cis-sabinene hydrate	1065	1069	0.04	0.01
**21**	terpinolene	1083	1086	1.16	0.08
**22**	p-cymenene	1087	1089	0.09	0.01
**23**	linalool	1090	1095	0.17	0.01
**24**	isopentyl isovalerate	1101	1102	0.09	0.01
**25**	β-thujone	1117	1118	0.02	0.00
**26**	cis-p-menth-2-en-1-ol	1120	1124	0.07	0.01
**27**	trans-pinocarveol	1139	1141	0.16	0.02
**28**	trans-verbenol	1144	1145	0.06	0.01
**29**	isoborneol	1163	1165	0.08	0.01
**30**	terpinen-4-ol	1171	1174	2.97	0.14
**31**	p-cymen-8-ol	1178	1179	0.11	0.01
**32**	α-terpineol	1183	1186	0.37	0.03
**33**	verbenone	1201	1204	0.07	0.01

**34**	trans-carveol	1214	1215	0.03	0.01
**35**	citronellol	1220	1223	0.02	0.01
**36**	methyl citronellate	1255	1257	0.07	0.01
**37**	isobornyl acetate	1279	1283	0.33	0.02
**38**	2-undecanone	1291	1293	0.05	0.01
**39**	α-terpinyl acetate	1344	1346	0.05	0.01
**40**	α-cubebene	1342	1345	0.47	0.04
**41**	α-copaene	1372	1374	0.38	0.02
**42**	β-elemene	1388	1389	0.78	0.05
**43**	sibirene	1398	1400	0.24	0.01
**44**	longifolene	1406	1407	0.04	0.01
**45**	(E)-caryophyllene	1415	1417	1.89	0.13
**46**	γ-elemene	1430	1432	0.14	0.01
**47**	β-copaene	1428	1430	0.08	0.01
**48**	cis-thujopsene	1432	1433	0.08	0.01
**49**	(E)–β–farnesene	1452	1452	0.38	0.03
**50**	α-humulene	1454	1454	1.79	0.09
**51**	trans-cadina-1(6),4-diene	1472	1475	0.10	0.02
**52**	γ-muurolene	1478	1478	0.44	0.04
**53**	germacrene D	1481	1480	2.04	0.10
**54**	valencene	1493	1492	0.23	0.03
**55**	bicyclogermacrene	1501	1500	0.64	0.06
**56**	β-bisabolene	1507	1505	0.09	0.01
**57**	γ-cadinene	1515	1512	0.34	0.02
**58**	δ-cadinene	1520	1518	1.20	0.12
**59**	selina-4(15),7(11)-diene	1542	1540	0.11	0.02
**60**	selina-3,7(11)-diene	1546	1545	0.10	0.01
**61**	(E)-nerolidol	1563	1561	0.09	0.01
**62**	germacrene B	1565	1559	0.79	0.06
**63**	spathulenol	1580	1577	0.09	0.01
**64**	caryophyllene oxide	1583	1582	0.20	0.03
**65**	humulene epoxide II	1610	1608	0.10	0.01
**66**	1-epicubenol	1627	1627	0.04	0.01
**67**	τ-muurolol	1641	1640	0.11	0.02
**68**	α-cadinol	1652	1652	0.12	0.03
	**TOTAL**			**98.22**	**1.80**

(d) *Origanum vulgare* L.

**Peak#**	**Compound**	**RI $_{exp}$**	**RI $_{pub}$**	**Area (%) Mean (n = 3)**	**std.dev**
**1**	methyl 2-methylbutyrate	768	769	0.10	0.02
**2**	(3Z)-hexenol	850	853	0.01	0.01
**3**	3-heptanone	886	885	0.01	0.01
**4**	tricyclene	920	921	0.01	0.01
**5**	α-thujene	923	924	1.81	0.02

**6**	α-pinene	932	932	0.74	0.07
**7**	camphene	947	946	0.18	0.01
**8**	sabinene	970	972	1.43	0.12
**9**	1-octen-3-ol	980	978	0.59	0.03
**10**	3-octanone	986	986	0.11	0.02
**11**	myrcene	990	991	1.97	0.08
**12**	3-octanol	997	999	0.02	0.01
**13**	α-phellandrene	1001	1002	0.16	0.02
**14**	δ-3-carene	1005	1008	0.05	0.00
**15**	α-terpinene	1015	1018	1.23	0.12
**16**	p-cymene	1023	1025	11.06	0.34
**17**	limonene	1029	1030	0.21	0.04
**18**	β-phellandrene	1031	1031	0.16	0.03
**19**	(Z)-β-ocimene	1033	1032	0.87	0.11
**20**	(E)-β-ocimene	1043	1044	0.68	0.04
**21**	γ-terpinene	1054	1054	13.71	0.24
**22**	cis-sabinene hydrate	1065	1069	0.46	0.05
**23**	terpinolene	1083	1086	0.08	0.01
**24**	p-cymenene	1087	1089	0.02	0.00
**25**	linalool	1090	1095	0.26	0.03
**26**	trans-sabinene hydrate	1097	1098	0.18	0.02
**27**	cis-p-menth-2-en-1-ol	1115	1118	0.02	0.00
**28**	borneol	1170	1173	0.28	0.01
**29**	terpinen-4-ol	1171	1174	0.57	0.07
**30**	p-cymen-8-ol	1178	1179	0.01	0.00
**31**	α-terpineol	1183	1186	0.18	0.01
**32**	(Z)-dihydrocarvone	1210	1207	0.03	0.01
**33**	(E)-dihydrocarvone	1217	1215	0.02	0.00
**34**	carvacryl methyl ether	1243	1239	0.52	0.07
**35**	pulegone	1245	1241	0.02	0.01
**36**	carvone	1250	1246	0.03	0.01
**37**	thymol	1290	1289	1.13	0.14
**38**	carvacrol	1315	1317	56.43	0.57
**39**	α-cubebene	1342	1345	0.02	0.01
**40**	carvacrol acetate	1369	1370	0.03	0.01
**41**	α-copaene	1372	1374	0.03	0.01
**42**	β-bourbonene	1384	1382	0.08	0.01
**43**	β-elemene	1388	1389	0.03	0.01
**44**	(E)-caryophyllene	1415	1417	1.65	0.29
**45**	β-copaene	1428	1430	0.05	0.01
**46**	α-humulene	1454	1454	0.17	0.02
**47**	ε-muurolene	1455	1453	0.05	0.01
**48**	γ-muurolene	1481	1478	0.05	0.01
**49**	germacrene D	1481	1480	0.28	0.04
**50**	γ-amorphene	1496	1495	0.04	0.01
**51**	(E,E)-α-farnesene	1505	1505	0.39	0.04
**52**	β-bisabolene	1507	1505	0.60	0.04
**53**	γ-cadinene	1515	1512	0.07	0.01
**54**	δ-cadinene	1520	1518	0.17	0.03
**55**	(E)-α-bisabolene	1542	1540	0.04	0.01
**56**	spathulenol	1580	1577	0.12	0.04

**57**	caryophyllene oxide	1583	1582	0.20	0.03
**58**	humulene epoxide II	1610	1608	0.01	0.01
**59**	τ-muurolol	1641	1640	0.02	0.01
**60**	α-cadinol	1652	1652	0.03	0.01
	**TOTAL**			**99.34**	**0.30**
